# Adoptive Immunotherapy and High-Risk Myeloma

**DOI:** 10.3390/cancers15092633

**Published:** 2023-05-06

**Authors:** Catherine Duane, Michael O’Dwyer, Siobhan Glavey

**Affiliations:** 1 Department of Haematology, Beaumont Hospital, D09 V2N0 Dublin, Ireland; 2 Department of Haematology, University of Galway, H91 TK33 Galway, Ireland; 3 Department of Pathology, Royal College of Surgeons in Ireland, D09 V2N0 Dublin, Ireland

**Keywords:** multiple myeloma, high-risk multiple myeloma, CAR-T cell therapy, TCR therapy, adoptive cellular therapy

## Abstract

**Simple Summary:**

Multiple myeloma is a cancer of the bone marrow which accounts for approximately 1.8% of all cancer diagnoses worldwide. The clinical outcomes for patients with multiple myeloma have significantly improved in the most recent decade, resulting in better quality of life and prolonged survival. However, this remains a largely incurable disease with inevitable relapse and resistance to medical treatment. Furthermore, there is a cohort of high-risk myeloma patients who possess disease characteristics which predispose them to worse clinical outcomes and poor response to treatment. There is a pressing need to develop new treatment strategies for myeloma, particularly therapeutic approaches which would be successful in high-risk patients. Recently there has been promising advances in cell-based immunological therapies for myeloma; however, further studies are needed to fully establish their long-term efficacy and their role in the treatment of high-risk patients.

**Abstract:**

Despite significant improvements in the treatment of multiple myeloma (MM), it remains mostly incurable, highlighting a need for new therapeutic approaches. Patients with high-risk disease characteristics have a particularly poor prognosis and limited response to current frontline therapies. The recent development of immunotherapeutic strategies, particularly T cell-based agents have changed the treatment landscape for patients with relapsed and refractory disease. Adoptive cellular therapies include chimeric antigen receptor (CAR) T cells, which have emerged as a highly promising therapy, particularly for patients with refractory disease. Other adoptive cellular approaches currently in trials include T cell receptor-based therapy (TCR), and the expansion of CAR technology to natural killer (NK) cells. In this review we explore the emerging therapeutic field of adoptive cellular therapy for MM, with a particular focus on the clinical impact of these therapies for patients with high-risk myeloma.

## 1. Introduction

Multiple myeloma (MM) is a haematological malignancy characterised by clonal proliferation of plasma cells in the bone marrow [1]. MM demonstrates a variable clinical course with alternating periods of remission and treatment response with relapse and disease progression. MM is the second most common haematological malignancy, accounting for approximately 1.8% of all new cancer diagnoses worldwide and 2.9% of global cancer deaths [2].

While MM remains an incurable malignancy, there have been significant advances in treatment in recent decades, resulting in vastly improved survival rates [1]. The worldwide estimated relative survival at 5 years is 57.9%, as calculated between 2012 and 2018 [2]. However, the survival rate for patients with high-risk multiple myeloma (HRMM) is significantly worse than standard-risk MM, with a 5-year overall survival (OS) of approximately 40%, compared to 82% in those without high-risk features [2].

The introduction of proteasome inhibitors (PIs) and immunomodulatory drugs (IMiDs) led to a dramatic improvement in clinical outcomes for MM, which were further enhanced by the development of monoclonal antibody therapies targeting CD38 and SLAMF7 [3,4]. The introduction of antibody therapies provided new therapeutic options for relapsed and refractory disease, thus lengthening overall survival. However, MM is a heterogenous disease with a highly variable clinical outcome, and the relatively recent improvement in overall survival has not been consistent across the patient population [5]. This variable clinical course reflects the diverse molecular profile of MM and the category of patients with HRMM [2]. Furthermore, the clonal evolution of MM under the selective pressure of treatment has been shown to occur frequently, which may contribute to disease progression and resistance to therapy [6]. Despite therapeutic advances, patients with unfavourable prognostic markers, such as high-risk cytogenetics, a high number of circulating plasma cells, and extramedullary disease remain poorly responsive to treatment [5,7]. There is a need for personalised, risk-adjusted treatment approaches for MM in order to maximise therapeutic benefit and reduce toxicity. 

In recent years, advanced treatments including bispecific antibodies and cellular immunotherapies such as chimeric antigen receptor T cell therapy (CAR-T), and T cell receptor therapy (TCR-T) have been developed. Patients with high-risk features that have not attained the same therapeutic benefits of recent frontline regimens require innovative treatment strategies to improve the clinical outcomes in this difficult-to-treat cohort. Cellular therapies have shown great success in the management of haematological malignancies, including B cell leukaemia and lymphoma using CAR-T therapy [8,9,10]. Adoptive cell therapies for MM include synthetic CAR T-cells directed at myeloma cell targets and the newer T cell receptor (TCR) therapeutic strategies. The application of the CAR therapeutic construct to other immune cells, including natural killer cells (CAR-NK), presents a strong therapeutic candidate, and is currently undergoing further investigation [11]. In MM, CAR-T therapy has demonstrated impressive success in treatment of relapsed or refractory patients. However, due to a variety of factors, many patients still experience early relapse following treatment, and the role played by disease risk status in relapse has not yet been fully characterised. Strategies for overcoming CAR-T resistance mechanisms and increasing therapeutic durability are needed, particularly in relation to HRMM. This review aims to explore the role of novel adoptive immunotherapies in the treatment of HRMM.

## 2. High-Risk Multiple Myeloma

The concept of high-risk MM has evolved in the past decade to incorporate specific prognostic factors including both cytogenetic and clinical biomarkers. Several risk stratification tools have been developed with the aim of identifying patients with high-risk disease. The Revised International Staging System (R-ISS) was established in 2015 to improve the specificity of risk stratification of patients, compared to the standard International Staging System (ISS). The revised tool incorporates two additional prognostic factors: lactate dehydrogenase (LDH) level and cytogenetic risk, identifying three specific high-risk chromosomal abnormalities: t(4;14), t(14;16), and del17p [2]. The inclusion of specific cytogenetics followed the publication of large-scale studies, consistently linking their presence with a poor prognosis [12,13]. The R-ISS was recently further updated to include the independent poor prognostic factors of 1q gain (three copies of 1q) or amplification (≥ four copies), as part of R2-ISS [14]. The presence of two high-risk factors is considered double-hit myeloma and the presence of three or more high-risk factors is triple-hit myeloma. However, even within the genetically defined high-risk subgroups, there is outcome heterogeneity, suggesting the presence of other factors in disease evolution and the need to further expand the risk stratification of patients [15]. Aside from the features included in the R-ISS/R2-ISS, several other biological and clinical prognostic factors have been identified which could further inform the definition of HRMM, such as the presence of extra-medullary disease and high levels of circulating plasma cells (≥5 cells/μL), both of which are independent poor prognostic factors [16,17,18]. Frailty status, as determined by the International Myeloma Working Group (IMWG) scoring system, is also an independent predictor of overall survival [19]. In addition to genetic and clinical parameters of risk status, there is also a cohort of patients who are refractory to induction therapy or experience early relapse within 12 months, who do not possess the established markers of high-risk disease; these patients are classified as functional high risk (FHR) and typically demonstrate increased mutations affecting the IL-6/JAK/STAT3 pathway [20,21]. These patients represent a further therapeutic challenge, given their limited response to frontline therapy. 

Almost all patients with MM eventually relapse with disease progression. Of note, 10–20% of patients experience early death within 2 years of diagnosis and these cases are generally patients with HRMM, such as those with extramedullary disease (EMD) or high-risk cytogenetic abnormalities (CAs) including t(4;14), t(14;16), t(14;20), gain (1q), del (17p), and TP53 mutations [22,23,24]. Indeed, HRMM patients have significantly poorer responses to treatment and overall clinical outcomes [22]. Thus, there is a need to develop novel therapeutic strategies and risk-stratified guidelines for MM, with particular consideration of frontline and rescue treatment options for patients with HRMM.

### Therapeutic Advances in High-Risk Multiple Myeloma

Newly diagnosed patients with HRMM represent a therapeutic challenge. This patient population generally has a poor prognosis with standard first-line therapy, experiencing earlier relapse, disease progression, and death, compared with standard-risk patients. There are a limited number of first-line phase III trials focused entirely on HRMM. Furthermore, the number of high-risk patients included generally represents a relatively small proportion, typically <20%, with most trials performing retrospective (planned or post hoc) analysis of high-risk subgroups, rather than stratifying patients by risk group at trial commencement [25,26,27]. Developing effective treatment options for patients with HRMM at diagnosis and relapse remains a significant challenge and an area of unmet clinical need [28,29,30]. Several studies have advocated for an adjusted treatment regimen for high-risk patients, including intensification of frontline therapy, altering the sequence of treatments, and introducing cellular therapy at an earlier stage [31]. Current recommendations for HRMM include the International Myeloma Working Group (IMWG) recommended triplet induction therapy with a PI, lenalidomide, and dexamethasone, followed by ASCT [13]. The ongoing GMMG-CONCEPT trial (NCT03104842) evaluates an intensified quadruplet regimen of isatuximab, carfilzomib, lenalidomide, and dexamethasone (Isa-KRd) as a frontline therapy in HRMM [32]. Interim results from this trial demonstrated favourable outcomes with regard to early response rates and two-year progression-free survival (PFS) associated with this intensified frontline regimen in high-risk patients. Notably, however, the SWOG 1211 trial (NCT01668719) evaluating quadruplet therapy in HRMM patients, involving the addition of anti-SLAMF7 monoclonal antibody elotuzumab to bortezomib, lenalidomide, and dexamethasone (RVd-elotouzumab), did not produce improved clinical outcomes associated with the addition of the fourth agent [33]. The ongoing OPTIMUM/MUKnine (NCT03188172) trial, evaluates the efficacy of daratumumab in combination with bortezomib, lenalidomide, cyclosphosphamide, and dexamethasone (Dara-CVRd) as an induction therapy in patients with ultra-high-risk disease (UHiRMM) (≥2 high-risk genetic lesions or plasma cell leukaemia) [34]. Additionally, this trial evaluated an intensification of consolidation and maintenance therapy with the addition of daratumumab to these regimens in the UHiRMM cohort. Dara-VRd was continued post-ASCT for 18 cycles, followed by Dara-R maintenance therapy. Interim results demonstrated favourable PFS in this cohort (77% at 30 months) when compared to the Myeloma XI (39.8% at 30 months) and MASTER trials (58% at 24 months) [35,36].

Improved understanding of the bone marrow and immunological microenvironment have paved the way for the development of highly effective immunotherapies, including bispecific antibodies and T-cell engagers, as well as adoptive cellular therapies [37,38]. Cellular therapies, including CAR-T and TCR therapies, have demonstrated promising clinical response rates in relapsed or refractory myeloma (R/RMM). However, there is growing interest in the application of these novel treatment strategies in the frontline therapy setting for patients with HRMM.

## 3. Adoptive Immunotherapy for Multiple Myeloma

Adoptive cellular immunotherapies represent an emerging field of treatment options for patients with R/RMM and particularly patients with HRMM. As the body of evidence grows for the deployment of advanced cell-based therapies, such as CAR-T therapies and TCR-based therapies, their role in the treatment of patients with HRMM is of particular interest. Several adoptive therapies have been approved for clinical use, with others currently under active investigation in clinical trials. To date, two CAR-T cell products, idecabtagene vicleucel (ide-cel) and ciltacabtagene autoleucel (ciltacel), have been approved for the treatment of R/RMM. There are several other CAR-T products in clinical trials at present (Table 1 and Table 2), as well as TCR therapies and cutting-edge CAR-NK cell therapy products.

There are several challenges associated with adoptive immunotherapy, including the complexity of the manufacturing process, prolonged vein-to-vein time, adverse events, and treatment resistance mechanisms. Adverse events associated with T-cell adoptive therapies have been well described in the literature [39,40,41]. This is particularly significant for CAR-T therapy where extensive cytokine release frequently results in Cytokine Release Syndrome [41]. CRS is an inflammatory state triggered by the release of cytokines and chemokines [42]. Other notable adverse outcomes are immune effector cell-associated neurotoxicity syndrome (ICANS), cytopenia, and infections [43,44].

## 4. CAR-T Cell Therapy in Multiple Myeloma

Chimeric antigen receptor T-cell (CAR T-cell) therapy has emerged in recent years as a promising immunotherapy for haematological malignancies, with several treatments currently licensed for relapsed refractory MM. CAR T-cells targeting specific tumour surface antigens are generated using T cells from the patient which are genetically modified to incorporate a specific tumour-targeting receptor. The chimeric antigen receptors are recombinant structures where the binding site of a monoclonal antibody is fused to intracellular signalling molecules and a co-stimulatory domain, such as the 4-1BB molecule in the two approved CAR-T products [44,45]. Within the standard CAR structure there is a single chain variable fragment (scFv), comprised of immunoglobulin heavy and light chain variable regions, resulting in recognition of tumour surface antigens without MHC-restricted antigen presentation [45]. Not all CARs employ an scFv; for example, some use ligands and some utilise heavy chain only. Thus, CARs are comprised of an antigen recognition domain and an intracellular T cell activation domain, conferring dual-signalling capabilities, facilitating T cell expansion and activation upon repeated exposure to the target antigen [46]. Following successful development of CAR-T therapies targeting CD19 for refractory non-Hodgkin’s B-cell lymphoma (B-NHL) and B-cell acute lymphoblastic leukaemia (B-ALL), a CAR T-cell therapy targeting B-cell maturation antigen (BCMA) in MM was developed [47,48]. There are currently two anti-BCMA CAR-T therapies licensed for treatment of relapsed refractory MM: idecabtagene vicleucel (ide-cel) and ciltacabtagene autoleucel (ciltacel). The major adverse effects associated with CAR-T therapy are cytokine release syndrome (CRS), immune effector cell neurotoxicity syndrome (ICANS), and cytopenias [49]. These toxicities may limit the widespread adoption of this therapy, particularly in frail patients.

BCMA is a transmembrane glycoprotein expressed on plasma cells and is a member of the tumour necrosis factor (TNF) receptor superfamily [50]. BCMA is involved in promoting the survival of long-lived plasma cells [51,52]. BCMA is expressed on plasma cells, both normal and clonal, but not on haematopoietic stem cells or non-haematological cells [53]. Notably, BCMA is highly expressed on MM cells and its expression is maintained through relapse, extramedullary spread, and residual disease post-therapy [54]. Anti-BCMA CAR T-cell therapy has been shown to elicit a therapeutic response, improving OS and PFS in patients with R/RMM [47,55,56]. Patients with extramedullary disease, a cohort with an invariably poor prognosis, also benefited from anti-BCMA CAR-T therapy, although these patients generally had a shorter PFS (121 days vs. 361 days) and OS (248 days vs. 1024 days) compared to those without extramedullary disease [57]. Of note, 45% of patients in this study had high-risk cytogenetic abnormalities, three patients with extramedullary disease and six patients without. Despite the favourable clinical outcomes associated with anti-BCMA CAR-T therapy, the therapeutic response is often short-term with disease relapse and progression in many patients, and this is particularly prevalent among HRMM patients [47,58]. Alternative CAR-T therapeutic targets are currently being investigated, including GPRC5D [59,60]. Furthermore, there is ongoing research into strategies to overcome resistance mechanisms in order to improve the durability of the response to CAR-T therapy, particularly in HRMM patients who have a significantly shorter PFS and OS post-CAR-T [58].

**Table 1:** Selection of landmark trials for CAR-T therapy in MM. Abbreviations: HR-CA, high-risk cytogenetic abnormalities; EMD, extramedullary disease; NR, not reached; CRS, cytokine release syndrome; ICANS, immune effector cell-associated neurotoxicity syndrome.

Trial Number/Name	Phase	CAR Construct	Target	Number of Patients at Last Update	Disease Risk Status: High-Risk Cytogenetics/Extramedullary Disease (%)	ORR (%)	Median PFS (Months)	Adverse Events: Grade ≥3 CRS/ICANs (%)	Ref
NCT03361748/KarMMa	I/II	Bb2121/Ide-cel	BCMA	140	HR-CA: 35% EMD: 39%	73%	8.8	CRS 5% ICANS 3%	[61]
NCT03430011/EVOLVE	I/II	JCARH125/Orva-cel	BCMA	115	HR-CA 41%	91%	NR	CRS 2% ICANS 4%	[62]
NCT03548207/CARTITUDE-1	I/II	JNJ-68284528/ LCAR-B38M/ Ciltacabtagene autoleucel (Cilta-cel)	BCMA	97	HR-CA 24% EMD: 13%	97%	NR	CRS 5% ICANS 9%	[63]
NCT03274219/CRB-402	I/II	bb21217	BCMA	72	Data unavailable	69%	NR	CRS 4% ICANS 7%	[64]
NCT04133636/CARTITUDE-2	III	JNJ-68284528 /LCAR-B38M/ Ciltacabtageneautoleucel (Cilta-cel)	BCMA	20	HR-CA: 15% EMD: 25%	95%	NR	CRS 10% ICANS 0%	[65]

### 4.1. CAR-T Therapy, Resistance Mechanisms, and High-Risk Patients

CAR-T therapy is complex, expensive, and is associated with significant risk of adverse events with variable durability regarding clinical response and outcomes. Thus, optimising patient selection is an important consideration. Disease risk status has been demonstrated as an important factor to inform the correct selection of therapy for patients with MM.

Patients with HRMM generally have a poor prognosis with current first-line therapeutic options, highlighting an unmet therapeutic need for this subgroup. The results from the ongoing CARTITUDE-2 study (NCT4133636) cohort B, which enrolled functional high-risk patients (progression ≤12 months after ASCT or ≤12 months after start of anti-myeloma therapy), demonstrated early and deep clinical responses to cilta-cel CAR-T therapy [65]. A recently published meta-analysis of 17 trials, involving a cumulative 723 patients, evaluated the evidence for CAR-T therapy in patients with high-risk disease, defined as the presence of high-risk cytogenetics, EMD, or an R-ISS stage III [58]. ORR and MRD were the main efficacy outcomes used. A high-risk cytogenetic profile was associated with a significantly worse ORR, when compared to standard-risk genetics (*p* = 0.01). Furthermore, the presence of high-risk cytogenetics was associated with a worse outcome in terms of PFS (*p* < 0.001) and an increased risk of MRD positivity, although this was not statistically significant (*p* = 0.06). Overall, it was concluded that high-risk cytogenetics were associated with a 70% increased risk of progression, relapse, or death. Interestingly, EMD was not associated with a significantly worse ORR following CAR-T therapy (*p* = 0.26). With regard to PFS and OS, however, the presence of EMD was associated with a significantly worse outcome. The 2-year follow-up of the CARTITUDE-1 trial, a phase Ib/II study evaluating the safety and efficacy of cilta-cel in heavily pre-treated patients with R/RMM, found that deep and durable responses were maintained at 2 years; however, the duration of response, PFS, and/or OS were lower in the high-risk subgroup [63]. Similarly, Hansen et al. recently published a study documenting real-world experience with ide-cel which also demonstrated an inferior PFS in patients with HRMM [66]. It would be expected that patients with a baseline poorer prognosis and higher risk disease would have worse outcomes following therapy than standard-risk patients; however, assessing the efficacy of treatments in this patient subgroup is a fundamental step towards risk-adjusted clinical management. It is likely that the reason for worse clinical responses to CAR-T therapy is multi-factorial and underscores the importance of understanding and overcoming resistance to CAR-T therapy. Possible resistance mechanisms are linked to antigen escape, CAR-T cell exhaustion, and the complex tumour microenvironment in MM, an issue of even greater significance in HRMM patients [67,68,69,70].

#### 4.1.1. Antigen Escape and Dual-Targeting CAR-T Cells

Patients with high-risk cytogenetics and EMD have a significantly worse response to CAR-T therapy with regard to PFS and OS and thus further investigation of resistance mechanisms and how they may be overcome is of critical importance in this subgroup. Antigen escape, as a result of downregulation or loss of the antigen under therapeutic pressure, is a potential source of resistance [67,71]. The role of antigen escape for BCMA CAR-T cell resistance has not yet been fully elucidated. While there is certainly a decline in BCMA expression following treatment with BCMA CAR-T cells, at the time of documented progression, patients often have significant residual BCMA expression on malignant plasma cells [72,73]. Notably, mutations in the BCMA epitope, preventing the binding of bispecific antibody therapy, have been described. It is unclear at present whether a similar process may play a role in CAR-T resistance, but in this setting, BCMA expression would remain detectable while the CAR would no longer be able to bind [74].

It is likely that the more genetically unstable the MM clones are, such as in HRMM, the greater the risk of clonal evolution resulting in antigen escape. There are a number of strategies under evaluation to overcome antigen escape mechanisms; in particular, the use of dual-targeted CAR-T cells that simultaneously target two antigens [75]. Several trials have combined CD38 or CD19 targeting mechanisms with BCMA, as a bispecific CAR-T therapy [76,77,78,79,80]. A recent phase II trial with 62 relapsed refractory patients evaluating a dual targeted anti-CD19 and BCMA CAR-T therapy demonstrated that this treatment had an ORR of 92% and induced a durable response with a median PFS of 18.3 months, with a 21.3 month median follow-up [81]. Of note, 29% of patients in this study had a high-risk cytogenetic profile and 24% had EMD. Interestingly, subgroup analyses in this study did not demonstrate any significant association between high-risk cytogenetics and PFS or OS post-therapy. However, patients with EMD had a significantly poorer PFS, with a median of 8.3 months versus 21.4 months in those without EMD. 

Other notable alternative targets for CAR-T therapy include SLAMF7 and GPRC5D [59,82]. Importantly, the results of recent small trials suggest that GPRC5D-targeting CAR-T cells demonstrated clinical efficacy in patients who were previously refractory for anti-BCMA CAR-T therapy [59,60], thus presenting a possible alternative pathway to overcome antigen escape in BCMA CAR-T therapy.

**Table 2:** Selection of dual-targeted CAR-T cell therapy trials in MM. Abbreviations: HRMM, high-risk MM; R/RMM, relapsed or refractory MM; ORR, overall response rate; MRD, minimal residual disease; PFS, progression-free survival; HR-CA, high-risk cytogenetic abnormalities; EMD, extramedullary disease.

Trial Number/Name	Phase	Dose CAR-T Cells per kg	Dual Targets	Number of Patients at Last Update	Disease Risk Status: High-Risk (%)	Clinical Response ORR MRD %	Median Follow-up (Months)	Adverse Events: Grade ≥3 CRS/ICANs (%)	Ref
ChiCTR1800018143	I	4 × 10^6^/kg	BCMA CD38	23 R/R MM	HR-CA 74% EMD 39%	ORR 87% MRD 87% PFS 17.2 months	9	CRS 17% ICANS 0%	[76]
ChiCTR1900026286	I/II	2.1 × 10^6^/kg	BCMA CD38	16 R/RMM	HR-CA EMD 50%	ORR 87.5% PFS rate 68.8% at 1 year	11.5	CRS 32% ICANS 0%	[77]
ChiCTR-OIC-17011272	II	1 × 10^6^/kg	BCMA CD19	62 R/RMM	HR-CA 24% R-ISS II/III 81%	ORR 92% MRD 77% PFS 18.3 months	21.3	CRS 10% ICANS 3%	[81]
NCT04935580	I/II	1 × 10^5^/kg 2 × 10^5^/kg 3 × 10^5^/kg	BCMA CD19	13 HRMM	100% patients had ≥1 high-risk feature (CA EMD IgD/IgE subtype, LDH > upper limit normal, R-ISS II/III	ORR 100% MRD 100%	5.3	CRS 0% ICANS 0%	[83]
ChiCTR1800017051	II	2 × 10^6^/kg	BCMA CD38	22 R/RMM	HR-CA 86.4% EMD 13.6% R-ISS II/III 100%	ORR 91% MRD 46% PFS rate 48.7% at 2 years	24	CRS 27% ICANS 0%	[84]

#### 4.1.2. CAR-T Exhaustion and the Tumour Microenvironment

CAR-T cell exhaustion, manifesting as dysfunction and impaired persistence, likely contributes to short-lived clinical remission and subsequent disease progression post-therapy [85,86]. The optimal persistence of CAR-T cells in MM is unknown and a lack of long-term persistence is not necessarily associated with a poor outcome. Of note in the CARTITUDE-1 trial, the favourable efficacy results for cilta-cel were achieved despite a lack of detectable CAR-T persistence in the peripheral blood [87]. The mechanisms underlying this therapeutic process are unclear; it is possible that CAR-T cells migrate to and persist at low levels in the bone marrow. Alternatively, if CAR-T cells do not persist and yet therapeutic response is observed, this suggests that highly active short-term immunotherapy can elicit a very effective clinical response and that short-term potency of CAR-T therapy, rather than long-term persistence, is the priority. 

The immunosuppressive tumour microenvironment, suboptimal T-cell function as a result of multiple previous lines of myeloma therapy, and persistent antigen stimulation are likely synergistic factors contributing to CAR-T cell exhaustion [85,88,89]. Optimising the CAR-T cell structure, including modification of co-stimulatory molecules and improving CAR-T effector function to alter the tumour immune microenvironment are potential strategies to reduce CAR-T exhaustion and thus improve the duration of the response to therapy [90,91].

The complex tumour microenvironment (TME) in MM is highly immunosuppressive, resulting from the increased expression of myeloid-derived suppressor cells (MDSCs), tumour-associated M2-like macrophages (M2 TAMs), N2 neutrophils, regulatory T cells (Tregs), regulatory B cells (Bregs), and plasmacytoid dendritic cells [92]. The dysregulated and immunosuppressed TME contributes to immune escape and resistance to immunotherapy for MM through upregulation of anti-apoptosis proteins, promotion of CAR-T cell exhaustion, and impairment of CAR-T cytotoxicity [70,93]. Overexpression of anti-apoptotic proteins, possibly mediated by adhesion to bone marrow stromal cells, results in MM cell resistance to CAR-T-mediated killing [94]. Furthermore, the upregulated expression of checkpoint inhibitors, such as programmed death-ligand 1 (PD-L1) by MM cells, further impairs immune activity by mediating T-cell exhaustion and impairing cytokine production [95]. It is likely that a cascade of events in the TME occur following failure of CAR-T cells to kill resistant MM cells, resulting in overactivation of CAR-T cells, which then induces an increase in immune checkpoint receptors such as PD-1, LAG-3, TIM3, and TIGIT, which can then be exploited by MM cells. It is possible that combination with immune checkpoint inhibitors could improve CAR-T function. An alternative strategy is to modify the CAR-T cells to disrupt the target immune checkpoint, such as PD-L1 [96,97]. Several other strategies to overcome the complex TME have been developed, including utilising cytokines and proinflammatory ligands to enhance CAR-T cell efficacy, as well as the combination of CAR-T therapy with an oncolytic virus [98,99,100]. Oncolytic viruses may be utilised in MM to transform the cold TME to hot, an approach which may be particularly beneficial to patients with TP53 deletions or mutations [101].

#### 4.1.3. CAR-T in Frontline Therapy for High-Risk Patients

Sequencing and stratification of therapeutic options based on disease risk status is a consideration in the management of MM, with several published and ongoing studies advocating for risk-adjusted frontline therapy. For example, a recent meta-analysis demonstrated that patients with high-risk cytogenetics benefited from the addition of the anti-CD38 monoclonal antibody Daratumumab as part of the backbone regimen in earlier lines of therapy, compared to standard-risk patients [30]. The conclusion from this meta-analysis was that patients with newly diagnosed HRMM may benefit from an altered, more intensive backbone regimen as the first line therapy and advocates for a risk-adjusted guideline for frontline therapy selection. The addition of Daratumumab to frontline induction, consolidation, and maintenance therapies for HRMM has been shown to improve MRD-negativity rates among high-risk patients [102]. The achievement of sustained MRD negativity may be considered a surrogate for longer PFS and OS, regardless of cytogenetic risk [103,104]. Thus, attainment of a durable MRD negativity may offset the poor prognosis associated with high-risk cytogenetics, emphasising the importance of frontline therapy selection. However, patients with high-risk disease are less likely to achieve MRD negativity than standard-risk patients and therefore may require intensification of their frontline therapy. Given the documented survival benefit from achieving and maintaining MRD negativity, the addition of CAR-T to frontline treatments for HRMM could increase the proportion of patients who achieve sustained MRD and thus improve prognosis. 

A small single-arm trial evaluating the efficacy and safety of a dual-target anti-CD19/BCMA CAR-T therapy post-ASCT as a frontline therapy for HRMM demonstrated an overall response rate (ORR) of 100% [79]. Notably, 70% of patients showed sustained minimal residual disease (MRD) for >2 years, with a median follow up of 42 months. Furthermore, the interim results of a larger ongoing trial evaluating an anti-BCMA/CD19 dual-targeting CAR-T therapy as a frontline therapy in newly diagnosed HRMM patients post-ASCT has demonstrated favourable results with 100% of patients achieving an overall clinical response (ORR), 69% achieving a stringent complete response (sCR), and 100% attaining MRD at a median follow-up of 5.3 months [83]. The results of these studies suggest that CAR-T therapy in earlier lines of treatment may induce deep responses in HRMM patients. It is possible that this altered sequencing of treatment strategy, including CAR-T therapy as a frontline treatment, could overcome the poor therapeutic response of this patient subgroup. In HRMM where baseline prognosis is poor, early application of adoptive immunotherapy at a time when there is a lighter disease burden and potentially greater bone marrow reserve may result in improved outcomes for this cohort. Furthermore, implementation of adoptive therapies at an earlier stage of treatment would reduce the consequences of heavy pre-treatment, resulting in a less compromised immune status and better T cell fitness, increasing the therapeutic benefit. The ongoing KarMMa-4 trial (NCT04196491) is a multi-centre, open-label study evaluating the use of CAR-T in HRMM patients (R-ISS III) who have received a maximum of three induction cycles [61]. The ongoing CARTITUDE-2 (NCT4133636) study which includes a cohort of functional high-risk patients (cohort B), 16% of whom also possess high-risk cytogenetics, is evaluating the efficacy and safety of CAR-T (cilta-cel) therapy in patients who received only one line of prior therapy [65]. The PFS at 12 months in this cohort was 90%, with MRD negativity achieved in 93% of patients. 

An important finding published in the Real-World Experience study by Hansen at al. was that patients with prior exposure to a BCMA-targeted therapy had lower response rates and shorter PFS [66]. Similarly, results published by Cohen et al. as part of CARTITUDE-2 (NCT04133636) demonstrated reduced clinical efficacy associated with cilta-cel treatment in patients with prior anti-BCMA therapy exposure [105]. Thus, consideration of previous lines of therapy is necessary with the aim of anti-BCMA therapy avoidance if CAR-T is planned, particularly in high-risk patients. The difficulty, however, is that patients with high-risk, aggressive disease may not have the time to wait for the complex manufacturing and administration of traditional autologous CAR-T product and may need to proceed with off-the-shelf anti-BCMA products. This clinical dilemma highlights the unmet need for an accelerated CAR-T manufacturing process such as FasTCAR-T (NCT04236011; NCT04182581), as well as off-the-shelf solutions such as CAR-NK cells.

### 4.2. CAR-NK Therapy for Multiple Myeloma

As outlined above, CAR-T therapy has seen significant advances and therapeutic success in recent years; however, limitations, including immune escape, CAR-T exhaustion, and adverse events, have prompted the exploration into the application of CAR technology to other immune cells, particularly natural killer (NK) cells. NK cells are the primary anti-tumour effector cells of the innate immune system and important mediators of antibody-dependent cellular cytotoxicity (ADCC) [106,107]. NK cells possess the ability to kill virally infected or malignant cells without prior immunologic encounter. Immune surveillance has a significant role in the development and progression of MM, with dysfunction and evasion of NK cells, in particular, playing a crucial role in disease progression and relapse [108,109].

NK cells are CD56-positive, CD3-negative, innate lymphoid cells that respond to virally infected or cancer cells with a variety of effector functions, mainly cell killing and production of pro-inflammatory cytokines. NK cells have a series of germ line-encoded activating and inhibitory receptors, and the activation status of NK cells is dependent on a delicate balance between activating signals on the one hand and inhibitory signals on the other. Loss of MHC class I on tumour cells leading to reduced inhibition of NK cells via KIR inhibitory receptors and/or recognition of stress-induced ligands on tumour cells may lead to the activation of NK cells. Once an NK cell commits to killing a target cell, it releases perforin and granzyme, inducing cell lysis. NK cells can also kill via the death receptor pathway, which is mediated by Fas ligand or TRAIL and the release of cytokines, such as IFN-gamma and TNF-alpha which are very important in promoting a subsequent adaptive immune response. Finally, in the presence of antibodies, engagement of CD16 leads to strong activation, which can over-ride other inhibitory signals leading to antibody-dependent cellular cytotoxicity (ADCC). NK cells are capable of innate recognition of myeloma cells via their activating receptors [110]. The administration of unmodified cord-derived NK cells following autologous stem cell transplant generated promising preliminary data, supporting the safety and potential efficacy of allogeneic NK cell transfer in patients with high-risk MM [111].

Several studies have demonstrated the anti-myeloma effects of NK cells, including in vitro studies which documented the ability of NK cells to recognise and initiate lysis in MM cell lines and primary patient myeloma cells [112,113]. Furthermore, it has been demonstrated that MM patients with a higher NK cell activity at presentation have a better cumulative survival, compared to those with low NK activity [114]. Thus, NK cells present a strong new therapeutic candidate for adoptive immunotherapy, possessing certain intrinsic properties that may overcome some limitations associated with T cell-based therapies. The therapeutic harnessing of NK cells for anti-tumour activity is not an entirely new concept in the MM treatment paradigm. Established therapeutics including immunomodulators (IMiDs), proteasome inhibitors (PIs), and monoclonal antibodies exert their anti-myeloma activity through engagement of immune function and the direct or indirect activation of NK cells [115]. 

Adoptive techniques generate large numbers of highly activated NK cells via ex vivo propagation strategies, which can then be re-infused to MM patients. CARs represent a promising platform for the delivery of adoptive NK therapy. It has been demonstrated that genetically modified CARs can direct NK cells towards malignant cell-specific killing, as well as retaining the CAR-independent mechanisms of NK-mediated cytotoxicity [116]. 

Allogeneic CAR-NK cell therapies could have many advantages over existing autologous CAR-T cell therapies. NK cells can recognize tumours in multiple different ways, potentially reducing the risk of failure due to antigen escape. NK cells are well tolerated with minimal risk of inducing CRS and ICANS toxicity. Unlike allogeneic T cells, NK cells do not cause GVHD and can be safely administered without the need for complex engineering [117,118]. CAR-T therapy requires the use of autologous T cells, which coupled with the complex manufacturing process prolongs the vein-to-vein time, a significant issue for high-risk patients with rapidly progressing disease in need of urgent treatment. Furthermore, as relapsed or refractory MM patients are generally heavily treated prior to CAR-T therapy, low T cell counts in the peripheral blood presents a challenge for autologous harvest [119]. Off-the-shelf NK cell therapies are immediately available, unlike autologous CAR T cells, benefiting patients with aggressive disease, in need of a rapid therapeutic response. Furthermore, batch manufacturing means that many patients can be treated by a single donor, markedly reducing costs. Indeed, as discussed above (Section 4.1.3), it now appears that prior treatment with a BCMA BSAB reduces the effectiveness of subsequent CAR-T cell therapy. In cases where treatment with CAR-T cells is the desired option, for example in HRMM patients, but where immediate treatment is required, CAR-NK cells could provide a bridge to subsequent CAR-T treatment, without compromising their efficacy, as might be the case following bispecific antibody treatment.

While clinical trials for CAR-NK are still ongoing, it appears that this therapy is well tolerated, with minimal risk of cytokine-mediated toxicity [120]. Pre-clinical studies evaluating CAR-NKs targeting MM antigens, including CD38, SLAMF7, and CD138, have demonstrated encouraging results [11,121,122] which warrant further development in early phase clinical trial studies. At present there are at least four active phase I clinical trials registered evaluating the safety and efficacy of BCMA-targeted CAR-NK cell therapy (NCT05008536, NCT05652530, NCT03940833, NCT05182073). 

## 5. T-Cell Receptor-Based (TCR) Adoptive Therapy

T-cell receptor (TCR)-based adoptive therapy utilises genetically modified T cells that are directed to bind specific tumour antigens [41]. TCR-T cells differ from CAR T-cells in structure and function, including a lack of co-stimulatory function and the ability of TCR-T cells to recognise peptide motifs bound to MHC, facilitating recognition of both surface and intracellular antigens [123]. Furthermore, TCR-T cells are also exquisitely more sensitive than CAR-T cells and can recognise antigens at a very low level. This mechanistic difference would suggest a possible advantage compared to CAR T-cells; however, this is counterbalanced by the risk of inefficacy upon MHC downregulation on tumour cells [124].

Antigen selection is a critical step in the TCR therapeutic process to avoid “on-target off-tumour” toxicity and adverse events. Cancer testis antigens (CTAs) are preferred targets for TCR-T, given that their expression in normal tissues is limited to germ cells, such as testes, which do not have HLA class I, thus reducing the risk of “on target” adverse events [125]. A phase I/II clinical trial of a TCR therapy recognising a naturally processed peptide shared by the cancer/testis antigens NY-ESO-1 and LAGE-1, was conducted in 20 antigen-positive MM patients with results published in 2015 by Rapoport et al. [126]. NY-ESO-1 and LAGE-1 were expressed in approximately 33% and 49% of MM patients retrospectively [124]. Of note, it has been previously demonstrated that expression of NY-ESO-1 in advanced MM correlated with high-risk cytogenetic features [127]. In this study by Rapoport et al., the patients received TCR therapy on day 1 post-autologous stem cell transplant (ASCT) and were then started on standard lenalidomide maintenance therapy. The authors reported that 70% of patients achieved near complete response (nCR) after treatment and the median progression-free survival (PFS) was 19.1 months (95% CI 8.5—not reached). Rapoport et al. described the clinical course of one patient with advanced refractory MM with extramedullary disease and high-risk cytogenetics (t(4;14)), following TCR therapy [126]. The patient attained a CR with normalisation of the bone marrow and clearance of extramedullary disease. The patient later relapsed, developing progression at a new extramedullary site; however, notably, the expression of NY-ESO-1 and LAGE-1 remained absent until the patient’s death at 2 years post-infusion, suggesting the role of ongoing immune surveillance and antigen escape in MM. 

A longer-term study (SPEAR-T) evaluating the efficacy and functional persistence of optimised TCR-T following ASCT was published by Stadtmauer and colleagues, with 52% of the cohort being progression-free at 1-year post-treatment [128]. The median PFS was 13.5 months (95% CI, 8.9–31.1 months) and median OS was 35.1 months (95% CI; 22.7 months not reached). Regarding the safety profile of TCR-T, this study reported that 52% of patients experienced serious adverse events (AEs), including neutropenia, autologous graft versus host disease, and atrial fibrillation. There were, however, no reports of cytokine release syndrome (CRS) and no adverse event reports consistent with neurotoxicity. There were no deaths attributed to adverse events in the study participants. Thus, in this small cohort, there was an acceptable safety profile associated with TCR therapy. A total of 48% of patients in this cohort had at least one cytogenetic abnormality, with one patient having a del17p; however, it is unclear if any patients had other high-risk chromosomal abnormalities such as t(4;14), or t(14;16). It is therefore difficult to comprehensively assess the impact of this treatment on patients with high-risk cytogenetics. Regarding long-term persistence of engineered T cells, functional studies demonstrated that 92% of the 25-patient cohort maintained quantifiable enhanced T-cells in the peripheral blood at day 100 post-infusion, with 40% at 1 year and 8% at 5 years post-infusion. Functionality was evaluated based on cytokine production. Correlation between persistence of engineered T cells, functionality, and clinical outcome was not ascertained due to the small sample size. This study demonstrated long-term persistence of engineered TCR T-cells up to 5 years post-infusion; however, the impact on clinical outcomes, such as PFS and OS, remain unclear due to the relatively small cohort size. Furthermore, given that patients received TCR therapy following ASCT and were receiving standard lenalidomide maintenance therapy, it is difficult to ascertain the relative contribution of the TCR therapy to the clinical outcomes. Notably, a phase I/II clinical trial (NCT01892293) with six enrolled participants evaluating a TCR targeting the same peptide in patients with relapsed refractory MM without concomitant ASCT was commenced in 2013. Only two of the six patients received the engineered T-cell therapy, and the study was terminated without publication of the data [129].

### TCR Therapy Resistance Mechanisms and High-Risk Patients 

In a similar manner to CAR-T, several likely mechanisms of resistance have been identified for TCR therapy, including downregulation of tumour antigen expression resulting in immune evasion, clonal selection pressure, T cell exhaustion, and lack of T-cell persistence [38]. Further modification of the TCR engineering process is ongoing in an effort to increase efficacy and prolong TCR therapeutic durability. In a 2020 study, Stadtmauer et al. further developed NY-ESO-1-TCR T-cells by knocking out the endogenous TCR α and β chains and PD-1 using CRISPR–Cas9 gene editing [96]. The rationale for this additional gene modification was to address the issue of persistently produced endogenous TCR α and β chains pairing with therapeutic TCR chains, likely resulting in reduced targeted TCR expression, as well as increased off-tumour adverse events [130,131]. Additionally, the knockout of immune checkpoint molecule PD-1 expression was designed to reduce T-cell exhaustion or dysfunction via this pathway [132]. The results of this study demonstrated extended persistence of the engineered T cells and in vitro experiments comparing the additional knockout T-cells to the NY-ESO-1 TCR transduced cells without additional gene modifications demonstrated higher antigen-specific cytotoxicity associated with the additional knockout products. Of note, only two MM patients were enrolled in this trial. Furthermore, while inclusion criteria required that all patients have a diagnosis of relapsed refractory MM with multiple prior lines of therapy, there was no record of the patients’ disease risk status on the basis of R-ISS and no outline of cytogenetic abnormalities.

The previously outlined studies demonstrate a possible role for TCR therapy in MM and an acceptable safety profile. However, there is a paucity of data in relation to TCR therapy for patients with high-risk disease, and this is the patient population with the greatest therapeutic need. The optimum timing of treatment with TCR therapy in patients with HRMM also requires evaluation. There is a need for further studies with a larger cohort size, including patients with high-risk cytogenetics and risk subgroup analyses, to evaluate the real-world clinical efficacy of TCR therapy, particularly in HRMM. 

## 6. Conclusions

In recent years, there has been significant advances in the development of immunotherapies for the treatment of MM. Important progress has been made in the design and application of adoptive cellular therapies for MM, including two licensed, FDA- and EMA-approved, CAR-T cell therapies. CAR-T has demonstrated impressive clinical results in patients with R/RMM; however, there remains several challenges with this treatment, including the complex manufacturing process, prolonged vein-to-vein time, and disease resistance and relapse. Other adoptive cellular therapies, currently in clinical trials, including TCR-therapy and more recently CAR-NK cell constructs, offer alternative therapeutic mechanisms and show conceptual attractiveness. These therapies may also offer a complimentary or synergistic approach for CAR-T therapy, if used in combination. Patients with HRMM have the most critical need for novel advanced therapeutic options; however, there is a paucity of data to inform treatment strategy for this specific subgroup. Further trials specifically designed to investigate and optimize this therapeutic approach in prospectively recruited high-risk patients, rather than solely as a subgroup analysis of larger studies, are needed [133]. The results of ongoing studies, such as the GMMG-CONCEPT and OPTIMUM/MUKnine trials, as outlined above will be useful in this regard. There is particular interest in the sequencing of therapeutic strategies in patients with HRMM and consideration of CAR-T in frontline treatment for this patient population is warranted, based on ongoing studies. Given that the prognosis worsens with each relapse and patients with HRMM are more likely to experience early relapse, there is a need for frontline treatments that provide disease control with durable responses and prolonged survival. Understanding the tumour microenvironment and factors that contribute to disease resistance, particularly in HRMM patients, will be crucial in the optimisation of established adoptive cellular therapies and development of the next generation of agents.
